# MicroRNAs and peritoneal dialysis

**DOI:** 10.1590/2175-8239-JBN-2026-E001en

**Published:** 2025-12-19

**Authors:** Hugo Abensur

**Affiliations:** 1 Universidade de São Paulo, Faculdade de Medicina, Hospital das Clínicas, São Paulo, SP, Brazil.; 2 A Beneficência Portuguesa de São Paulo, São Paulo, SP, Brazil.

MicroRNAs are small non-coding RNA molecules (19–24 nucleotides) that regulate post-transcriptional gene expression, mainly by inhibiting translation or degrading messenger RNAs (mRNAs). A single microRNA can target hundreds of mRNAs and influence the expression of many genes often involved in a functional interaction pathway. MicroRNAs can be isolated from cells, tissues, and body fluids such as serum, plasma, tears, or urine^1^.

Circulating microRNAs are being considered promising biomarkers for many human diseases, as they meet several criteria to be regarded as preferable biomarkers, including high specificity, easy accessibility, and sensitivity. The sensitivity of microRNAs has been demonstrated, and their levels can vary according to disease progression or response to treatment. With these advantages, miRNAs offer a non-invasive method for accurate diagnosis, disease progression prognosis, treatment guidance, and response assessment^2^.

The expanding knowledge about the role of microRNAs in biology and their dysregulation in many diseases has led scientists to explore their potential use in disease therapy. Two strategies have been employed: one as microRNA restoration therapy, where a negatively regulated or non-functional microRNA would be delivered via a synthetic oligonucleotide, and another as microRNA inhibition therapy, where overexpression of a microRNA would be inhibited by antagonists^3^.

The relationship between microRNAs and peritoneal dialysis primarily centers on regulating processes such as inflammation, fibrosis, and peritoneal membrane failure, which are common complications of peritoneal dialysis therapy^4^. For example, miR-21 is consistently associated with promoting peritoneal fibrosis, being found at elevated levels in patients undergoing peritoneal dialysis and correlating with membrane functional changes and episodes of peritonitis^5^. On the other hand, microRNAs like miR-132-3p^6^ and miR-15a-5p^7^ exhibit anti-fibrotic effects, inhibiting pro-fibrotic pathways and reducing inflammation, suggesting potential therapeutic use to prevent or treat dialysis-induced peritoneal fibrosis. Additionally, microRNA profiles in peritoneal dialysis effluent can serve as biomarkers to monitor membrane function, identify the risk of technique failure, and diagnose complications such as bacterial peritonitis^8^. Therefore, microRNAs play a central role in both pathogenesis as well as in the monitoring and potential treatment of complications associated with peritoneal dialysis.

In the study published in this edition of the Brazilian Journal of Nephrology, Costa et al.^9^ evaluated the expression of circulating microRNAs in patients with end-stage chronic kidney disease (CKD) on peritoneal dialysis (PD) and non-dialytic (ND) patients and determined the regulatory networks of the target genes of the discrepant microRNAs between PD and ND patients. Twenty adult patients were evaluated, 10 on PD for at least 3 months and 10 with stage 5 CKD not on dialysis. As a result, nine microRNAs showed dysregulated expression in the PD group compared to the ND group: eight were under expressed, and one was overexpressed. The results revealed 1,382 target genes regulated by all nine microRNAs. The genes regulated by microRNAs are mainly associated with GPCR signaling (which regulates blood flow, glomerular filtration, and tubular reabsorption processes), insulin resistance, and energy metabolism, playing roles in fibrosis and functions related to inflammation.

The assessment of microRNAs will soon become part of our clinical practice as biomarkers and therapeutic targets.

## Data Availability

No new data were generated or analyzed in this study.
