# Supplementary material for: MicroRNAs and peritoneal dialysis
Source: J Bras Nefrol. 2025 Dec 19;48(1):e2026E001. doi: 10.1590/2175-8239-JBN-2026-E001en (PMC12820990; doi:10.1590/2175-8239-JBN-2026-E001en)
Supplement: Translation

# Translation: MicroRNAs e diálise peritoneal

**DOI:** 10.1590/2175-8239-JBN-2026-E001pt

**Authors:** Hugo Abensur

**Affiliations:** 1 Universidade de São Paulo, Faculdade de Medicina, Hospital das Clínicas, São Paulo, SP, Brasil.; 2 A Beneficência Portuguesa de São Paulo, São Paulo, SP, Brasil.

Os microRNAs são pequenas moléculas de RNA não codificante (19–24 nucleotídeos) que regulam a expressão gênica pós-transcricional, principalmente por inibição da tradução ou degradação de RNAs mensageiros (mRNAs). Um único microRNA pode ter como alvo centenas de mRNAs e influenciar a expressão de muitos genes frequentemente envolvidos em uma via de interação funcional. Os microRNAs podem ser isolados de células, tecidos e fluidos corporais, como soro, plasma, lágrimas ou urina^1^.

Os microRNAs circulantes estão sendo considerados biomarcadores promissores para muitas doenças humanas, uma vez que atendem a vários critérios para serem considerados biomarcadores preferíveis, incluindo alta especificidade, fácil acessibilidade e sensibilidade. A sensibilidade dos microRNAs foi comprovada, e seus níveis podem variar de acordo com a progressão da doença ou com a resposta ao tratamento. Com essas vantagens, os mRNAs oferecem um método não invasivo para diagnóstico preciso, prognóstico da progressão da doença, orientação do tratamento e avaliação da resposta ao tratamento^2^.

A expansão do conhecimento sobre o papel dos microRNAs na biologia e sua desregulação em muitas doenças levou os cientistas a investigar seu potencial uso na terapia de doenças. Foram empregadas duas estratégias: uma como terapia de restauração de microRNA, na qual um microRNA regulado negativamente ou não funcional seria fornecido por um oligonucleotídeo sintético; outra como terapia de inibição de microRNA, na qual a superexpressão de um microRNA seria inibida por antagonistas^3^.

A relação entre microRNA e diálise peritoneal está centrada principalmente na regulação dos processos de inflamação, fibrose e falência da membrana peritoneal, que são complicações comuns da terapia de diálise peritoneal^4^. Por exemplo, o miR-21 está consistentemente associado à promoção da fibrose peritoneal, sendo encontrado em níveis elevados em pacientes submetidos à diálise peritoneal e correlacionando-se com alterações funcionais da membrana e episódios de peritonite^5^. Por outro lado, microRNAs como o miR-132-3p^6^ e o miR-15a-5p^7^ apresentam efeitos antifibróticos, inibindo vias pró-fibróticas e reduzindo a inflamação, sugerindo potencial terapêutico para prevenir ou tratar a fibrose peritoneal induzida pela diálise. Além disso, perfis de microRNA no efluente da diálise peritoneal podem servir como biomarcadores para monitorar a função da membrana, identificar risco de falência da técnica e diagnosticar complicações, como peritonite bacteriana^8^. Portanto, os microRNAs desempenham papel central tanto na patogênese quanto no monitoramento e no potencial tratamento das complicações associadas à diálise peritoneal.

No estudo publicado nesta edição do *Brazilian Journal of Nephrology*, Costa et al.^9^ avaliaram a expressão de microRNAs circulantes entre pacientes portadores de doença renal crônica (DRC) estágio 5 em programa de diálise peritoneal (DP) e não dialíticos (ND) e determinaram as redes regulatórias dos genes-alvo dos microRNAs discrepantes entre os pacientes em DP e ND. Foram avaliados 20 pacientes adultos, 10 em DP por pelo menos 3meses e 10 portadores de DRC estágio 5 ND. Como resultado, 9 microRNAs demonstraram expressão desregulada no grupo DP em comparação ao grupo ND: 8 estavam subexpressos e 1 estava superexpresso. Os resultados revelaram 1.382 genesalvo regulados por todos os 9 microRNAs. Os genes regulados por microRNAs estão principalmente associados à sinalização GPCR (regulação do fluxo sanguíneo, filtração glomerular e processos de reabsorção tubular), resistência à insulina e metabolismo energético, desempenhando papéis na fibrose e em funções associadas à inflamação.

A avaliação dos microRNAs, em breve, fará parte de nossa prática clínica como biomarcadores e alvos terapêuticos.

## Data Availability

Nenhum dado novo foi gerado ou analisado neste estudo.
